# A cross‐sectional survey on occupational stress and associated dyslipidemia among medical staff in tertiary public hospitals in Wenzhou, China

**DOI:** 10.1002/brb3.2014

**Published:** 2020-12-23

**Authors:** Hui Zhang, Meng‐Meng Shao, Xian‐Da Lin, Li‐Jun Cheng, Begench Ovlyakulov, Bo‐Bei Chen, Ke‐Yang Chen

**Affiliations:** ^1^ Department of Pediatric Allergy and Immunology The Second Affiliated Hospital and Yuying Children’s Hospital of Wenzhou Medical University Wenzhou China; ^2^ Department of Rehabilitation The First Affiliated Hospital of Wenzhou Medical University Wenzhou China; ^3^ Department of Neurology Wenzhou Peoples’ Hospital Wenzhou China; ^4^ Department of Otolaryngology The Second Affiliated Hospital and Yuying Children’s Hospital of Wenzhou Medical University Wenzhou China; ^5^ Department of Neurology The Second Affiliated Hospital and Yuying Children’s Hospital of Wenzhou Medical University Wenzhou China

**Keywords:** dyslipidemia, medical staff, occupational stress, relationship

## Abstract

**Introduction:**

Occupational stress is considered to be a harmful physical and emotional response to an individual's psychological and/or physiological state in the work environment and is highly prevalent among medical staff. However, few epidemiological studies have investigated occupational stress in medical staff. Our study aims to explore the characteristics of occupational stress and its relationship with dyslipidemia in Chinese medical staff at tertiary hospitals and establish the basis for future preventive strategies.

**Methods:**

A cross‐sectional study was conducted in three tertiary public hospitals in Wenzhou City, Zhejiang Province, China. Data were collected using random sampling procedures to examine demographic characteristics and job‐related data. The participants completed the Occupational Stress Inventory—Revised (OSI‐R) questionnaires and serum lipids tests. Partial correlation analysis was conducted to explore the relationship between occupational stress and dyslipidemia.

**Results:**

A total of 1,176 medical staff responses to questionnaires were obtained. The occupational stress levels of medical staff were higher than those of normative populations, while their coping resources were lower. Most of the subscales of occupational stress demonstrated higher results for doctors and males than for nurses and females with crude analyses. Each subscale of OSI‐R was found to be associated with a different type of blood lipid level.

**Conclusions:**

The occupational stress level of medical staff in tertiary public hospitals in Wenzhou was high, and occupational stress may contribute to dyslipidemia. An investigation into occupational stress levels and their association with dyslipidemia in this population could draw more attention to medical staff in tertiary public hospitals.

## Abstract 2

We explore the characteristics of occupational stress and relationships with dyslipidemia in Chinese medical staff at tertiary hospitals, to establish the basis for future preventive strategies.

## 1 INTRODUCTION

Stress is an important part of human social life. Appropriate pressure can help individuals overcome challenges and promote successful in their roles. However, overwhelming acute stress may adversely affect the sleep problem, fatigue (Philbert et al., 2011). Chronic stress exposures have been linked to early onset cardiovascular disease, hypertension, insulin resistance, anxiety, and depression (Herbison et al., 2017; Kivimäki & Kawachi, 2015). Occupational stress is considered to be a harmful physical and emotional response to an individual's psychological and/or physiological state in the work environment (Richardson & Rothstein, 2008). The commonly used evaluation methods of occupational stress were the Occupational Stress Inventory (OSI), the Job Content Questionnaire (JCQ) (Karasek et al., 1998), the Effort‐Reward Imbalance (ERI) questionnaire (Siegrist, 1996), the Job Stress Questionnaire (JSQ) (Kittel et al., 1983) and the Occupational Stress Questionnaire (OSQ) (Elo et al, 1992), and the Revised‐Edition of OSI was the most common used in China.

Occupational stress is recognized hazard in many professions (Schneider et al., 2015). However, due to different occupational characteristics, the stress evaluation models have different focuses and conclusions. Job stress is highly prevalent (Clough et al., 2017) in medical staff because they need to acquire medical knowledge and skills through long‐term, difficult training. Furthermore, their professional knowledge makes them responsible for the lives and health of others and due to the high intensity of the work itself.

Dyslipidemia refers to abnormal levels of lipids in the blood, usually represented as elevated total cholesterol (TC), triglyceride (TG), and/or low‐density lipoprotein cholesterol (LDL), as well as a decrease in high‐density lipoprotein cholesterol (HDL). Medical workers in Asia may have a higher prevalence of hypertension, dyslipidemia, and coronary artery disease (Chen et al., 2015; Kao et al., 2016; Tam et al., 2017). Many studies (Loerbroks et al., 2015; Nyberg et al., 2014; Tayama et al., 2016) have examined the relationship between occupational stress and obesity, hypertension, and type 2 diabetes mellitus, but only a few studies have focused on the relationship between occupational stress and dyslipidemia in Chinese medical staff. Previous studies have suggested that lipid disorders are related to job stress (Djindjić et al., 2013; Xu et al., 2011). However, the results of these studies are controversial, and the potential association between occupational stress and dyslipidemia has not been fully investigated. This study aimed to provide epidemiological data about occupational stress and its relationship with dyslipidemia and theoretical basis to assess their physical and mental health.

## 2 METHODS

### 2.1 Study design and participants

A cross‐sectional study was conducted among medical staff from the tertiary public hospitals in Wenzhou, Zhejiang, China between February 2018 and March 2019. Wenzhou is the third largest city in Zhejiang Province and includes three districts. The Cochran formula was employed to identify the sample size. The proportion of medium or high occupational stress level was considered to be 60% based on the results of pilot tests. With a desired confidence level of 95% and ± 5% precision, a sample size of 369 was obtained. The sample size calculation was increased to 1,106 to give better estimate of the population and minimize the effect of outliers. Subjects were selected through a two‐stage random sampling procedure. In the first stage of sampling, one tertiary A hospital was selected from one district using a computer‐generated random numbers table. In total, three of eight hospitals were included. In the second stage of sampling, participants in each hospital were selected based on doctors’ job numbers using another computer‐generated random number table. All participants were informed of the objectives and interests of the study and their right to refuse or agree to participate. They were invited to fill out an anonymous self‐administered questionnaire including demographic information, such as age, gender, education level, marital status, professional title, and their period of employment as medical staff in the hospital. We divided the time into “<10 years,” “10–20 years,” and “>20 years.” Physical activity per day was classified by exercise time into three levels: less than 30 min as level 1, 30 min–1 hr as level 2, and more than 1 hr as level 3. Body mass index (BMI) was calculated as weight in kilograms divided by the square of height in meters. The inclusion criteria for participation were as follows: clinical frontline staff (doctors and nurses) and (2) three years in current job title. The exclusion criteria were as follows: (1) collaborators/internship and rotation staff and (2) participants who took steroids, statins, contraceptive pills, and other medicines possibly related to dyslipidemia. The procedures were in accordance with the Declaration of Helsinki and the study was approved by the Ethical Committee of the Second Affiliated Hospital of Wenzhou Medical University.

### 2.2 Measurement of occupational stress

Occupational stress was assessed using the OSI‐R by Osipow (Osipow, 1998). The Chinese edition was initially adapted by Li (Li et al., 2001) to improve the value of the scale's application. The scale consists of three dimensions: the Occupational Role Questionnaire (ORQ), which includes six subitems consisting of role overload (RO), role insufficiency (RI), role ambiguity (RA), role boundary (RB), responsibility (R), physical environment (PE), with 10 entries for each subitem; the Personal Strain Questionnaire (PSQ) , which consists of four subitems, vocational strain (VS), psychological strain (PSY), interpersonal strain (IS), and physical strain (PHS), with 10 entries for each subitem; and the Personal Resources Questionnaire (PRQ) , which consists of four subitems, recreation (RE), self‐care (SC), social support (SS), and rational coping (RC); with 10 entries for each subitem, producing a total of 140 items. In the OSI‐R subscale, the higher the ORQ score, the heavier the task, and the higher the PSQ score, the greater the occupational stress. A higher PRQ score indicates that the respondent has a stronger ability to cope with occupational stress. In our sample, the Cronbach's *α* coefficients for the OSI‐R, ORQ, PSQ, and PRQ were 0.88, 0.87, 0.83, and 0.86, respectively. The OSI‐R subscales were classified into three grades: ORQ: low < 120, moderate 120–160, high > 160; PSQ and PRQ: low < 92, moderate 92–100, high > 100 (Luo et al., 2016).

### 2.3 Laboratory measurements

Fasting blood samples were obtained in the morning and centrifuged immediately for all participants. Biochemistry parameters were measured at the laboratory of our hospital. TC, TG, and HDL‐C were all determined using an Automatic Biochemistry Analyzer (Hitachi 7020). The concentration of LDL‐C was calculated using the Friedewald formula (Hedqvist & Moawad, 1975). The following lipid parameters were considered abnormal according to the 2016 Chinese Guideline for the Management of Dyslipidemia in Adults (Joint committee issued Chinese guideline for the management of dyslipidemia in adults, 2016): TG ≥ 2.26 mM; TC ≥ 6.22 mM; HDL‐C < 1.04 mM; LDL‐C ≥ 4.14 mM. Medical staff with any of these abnormalities or a previous diagnosis of dyslipidemia, or receiving lipid‐lowering therapy were classified as having dyslipidemia. All control values were consistent with the standards recommended by the medical laboratory of the China Centers for Disease Control and Prevention.

### 2.4 Statistical analyses

EpiData V 3.1 was used to establish the study's database. The following formula was used calculated the sample size: *N* = Z_1−α/2_^2^ *p*(1 – *p*)/*d* ^2^. 95% confidence interval (*Z*1 − *α*/2 = 1.96) and 5% absolute error (*d* = 0.05). To ensure accuracy, the data were checked by trained personnel after all surveys were completed and entered. The data were analyzed using SPSS 23.0 (SPSS—IBM, Incorporation). All statistical tests were two‐sided, and a *p* value < 0.05 was considered statistically significant. The one‐way analysis of variance (ANCOVA) with post hoc analysis was used to compare the means of the OSI‐R scores and blood lipid level in the demographic and job‐related data, and analysis of covariance adjusted for other factors. Multiple linear regression was performed to examine the associations between two variables whilst controlling for the effect of one or more other “control” variables.

## 3 RESULTS

In total, 1,367 participants met the criteria, but 191 medical staff were excluded from this study because of their unwillingness to participate. Of the participants, 1,176 returned complete questionnaires (valid response rate = 86.00%). Over half of the participants were women (59.70%). The average age was 38.52 ± 9.36 years for men and 36.61 ± 9.46 years for women. Furthermore, the prevalence of dyslipidemia was 59.00%, 72.10% of participants had an undergraduate degree or greater and 75.60% were married. Regarding occupational characteristics, the majority of the participants (70.30%) had rich work experience. The higher the position of the title, the fewer the number of participants (Table 1).

**TABLE 1:** Sociodemographic characteristics and general information of the participants (*N* = 1,176)

Variables	Number	Percentage
Gender		
Male	474	40.3
Female	702	59.7
Occupation		
Doctor	640	54.4
Nurse	536	45.6
Age group (years)		
<30	197	16.7
30–39	530	45.1
40–49	394	33.5
50–59	48	4.1
≥60	7	0.6
Education		
Junior college or under	356	27.9
Undergraduate	261	20.5
Graduate or above	658	51.6
Work experience		
<10	349	29.7
10–20	767	65.2
>20	60	5.1
Professional title		
Junior	439	37.3
Intermediate	561	47.7
Associate senior	136	11.6
Senior	40	3.4
Marital status		
Single	280	23.8
Married/cohabitation	889	75.6
Others	7	0.6
BMI		
BMI < 24	118	10
24 ≤ BMI<28	929	79
BMI ≥ 28	129	11
Physical activity		
Level 1	612	52
Level 2	463	39.4
Level 3	101	8.6

Among the participants in this study, the mean score of most ORQ and PSQ subscales in doctors and nurses were identified as statistically higher than Chinese normative samples (Yang et al., 2007), while those of the PRQ subscales were lower. Compared with nurses, doctors’ scores were higher on the ORQ and PSQ. Differences between OSI‐R subscale scores and the norm for male and female medical staff illustrated the same significant difference (*p* < .001), except for ORQ in males (*p = *.329). Male staff were found to have higher mean scores on the ORQ and PSQ and lower PRQ subscales than female staff (*p* < .05). There was no significant difference in OSI‐R scores for different titles (*p* > .05). Both doctors and nurses had higher ORQ and PSQ scores and lower PRQ scores (*p* < .001). Among the different departments, each subscale of OSI‐R scores were significantly different (*p* < .05) (Table 2). Internal had the lowest ORQ and highest PRQ scores after post‐hoc analysis (Table S1). PSQ was significantly different professional title, but no difference in ORQ and PRQ (Table 2).

**TABLE 2:** Level of Occupational Stress Inventory—revised edition (OSI‐R) by job category

Variable	ORQ	PSQ	PRQ
Gender			
Female	185.86 ± 19.57	105.58 ± 18.11	107.01 ± 20.12
Norm	158.50 ± 26.40	89.20 ± 16.90	129.60 ± 17.70
*t*	25.061	21.560	−27.879
*p*	<.001	<.001	<.001
Male	165.22 ± 20.56	96.94 ± 16.82	125.24 ± 19.47
Norm	166.50 ± 27.00	92.50 ± 17.30	128.90 ± 17.70
*t*	−0.977	5.121	−4.037
*p*	0.329	<0.001	<0.001
Occupation			
Doctor	180.91 ± 19.98	102.78 ± 18.87	115.96 ± 19.48
Norm	162.90 ± 27.00	91.00 ± 17.20	129.20 ± 17.70
*t*	16.223	15.950	−17.412
*p* Value	<.001	<.001	<.001
Nurse	171.17 ± 16.89	95.59 ± 20.12	118.85 ± 21.23
Norm	162.90 ± 27.00	91.00 ± 17.20	129.20 ± 17.70
*t*	6.923	5.708	−12.462
*p* Value	<.001	<.001	<.001
Department			
Internal	173.86 ± 19.39	100.67 ± 18.87	114.57 ± 18.32
Surgery	177.78 ± 17.54	102.78 ± 19.52	110.49 ± 21.46
Pediatrics	177.63 ± 19.09	102.98 ± 16.81	109.13 ± 18.76
Emergency	180.03 ± 19.58	104.91 ± 17.37	108.49 ± 19.11
*F*	8.29	3.85	9.18
*p* Value	<.001	.02	<.001
Professional title			
Junior	169.53 ± 16.29	98.67 ± 17.57	114.57 ± 16.34
Intermediate	172.89 ± 17.39	100.53 ± 19.12	112.96 ± 18.18
Associate senior	173.86 ± 19.53	103.67 ± 14.61	109.57 ± 20.13
Senior	174.53 ± 15.61	103.67 ± 18.87	108.38 ± 13.92
*F*	1.11	4.41	3.01
*p* Value	.428	.010	.063

Abbreviations: ORQ, Occupational Role Questionnaire; PRQ, Personal Resources Questionnaire; PSQ, Personal Strain Questionnaire.

The three levels of blood lipids in male medical staff were significantly higher than those in females (*p* < .05). The three levels of blood lipids of doctors were higher than those of nurses, and the difference was statistically significant (*p* < .05). As shown in Table 3, there was significant difference between the four different departments with regard to TC and TG (*p* < .05), but no significant difference with regard to HDL‐C and LDL‐C (*p* > .05). The triglycerides level of emergency department staff was the highest among four departments (*p* < .05). There were significant differences in TC and LDL‐C levels between the low‐, moderate‐, and high‐level occupational stress groups; conversely (Table S2), no statistically significant differences in HDL‐C and LDL‐C levels were observed.

**TABLE 3:** Comparison blood lipid level in different variable

Variable	TG	TC	HDL‐C	LDL‐C
Gender				
Male	1.86 ± 0.48	5.26 ± 0.86	1.26 ± 0.41	3.16 ± 0.77
Female	1.33 ± 0.30	4.83 ± 0.90	1.43 ± 0.45	2.66 ± 0.76
*t*	23.177	8.181	−6.584	11.018
*p*	<.001	<.001	<.001	<.001
Occupation				
Doctor	1.71 ± 0.47	5.19 ± 0.87	1.33 ± 0.43	3.07 ± 0.78
Nurse	1.37 ± 0.33	4.80 ± 0.91	1.45 ± 0.46	2.64 ± 0.76
*t*	14.090	7.497	−4.617	9.526
*p*	<.001	<.001	<.001	<.001
Department				
Internal	1.57 ± 0.95	4.99 ± 0.85	1.35 ± 0.33	2.86 ± 0.71
Surgery	1.60 ± 0.94	5.10 ± 0.91	1.32 ± 0.34	2.89 ± 0.78
Pediatrics	1.45 ± 0.39	4.82 ± 0.87	1.47 ± 0.46	2.78 ± 0.75
Emergency	1.87 ± 0.47^a^	5.05 ± 0.97	1.40 ± 0.35	2.87 ± 0.82
*F*	47.57	15.42	1.43	2.86
*p*	.001	.001	.066	.057
OSI‐R group				
Low OSI‐R	1.33 ± 0.53	4.61 ± 0.85	1.43 ± 0.42	2.73 ± 0.68
Moderate OSI‐R	1.72 ± 0.49	5.18 ± 1.54	1.32 ± 0.21	2.56 ± 0.64
High OSI‐R	1.74 ± 0.39	5.89 ± 1.13^*^	1.29 ± 0.28	2.35 ± 0.59
*F*	77.36	75.67	21.38	34.86
*p*	.051	<.001	.089	<.001

Compared with internal, surgery, pediatrics, emergency was the highest by post hoc analysis. Compared with low‐, moderate group, high OSI‐R group was the highest in TC by post hoc analysis. ^*^ *P<0.05*

Abbreviations: HDL‐C, high‐density lipoprotein cholesterol; LDL‐C, low‐density lipoprotein cholesterol; TC, total cholesterol; TG, triglyceride.

Table 4 lists the correlations of the ORQ, PSQ, and PRQ scores with TC, TG, HDL‐C and LDL‐C. The LDL‐C level of medical staff was statistically significantly positively correlated with the ORQ, PSQ and PRQ. Two items of the serum lipid profile, including TG, TC, were positively correlated with ORQ and PSQ without adjustment (*p* < .05). After controlling for age, education, BMI, smoking, alcohol consumption and physical activity, only TC correlated with ORQ, PSQ (*p* < .001). However, HDL levels were negatively correlated with the ORQ, PSQ, and PRQ scores after adjustment (*p* < .001). PRQ had only weakly significant correlations after adjustments.

**TABLE 4:** The correlations of subscale OSI score with the serum lipid level in medical staff

	ORQ			PSQ			PRQ		
	Model	*β* (95%CI)	*p*	Model	*β* (95%CI)	*p*	Model	*β* (95%CI)	*p*
Triglycerides	a	0.561 [0.348, 0.621]	<.001	a	0.311 [0.283, 0.361]	.036	a	−0.265 [−0.336,−0.145]	.331
	b	0.449 [0.394, 0.510]	.069	b	0.226 [0.175, 0.265]	.721	b	−0.254 [−0.325,−0.132]	.452
Total cholesterol	a	0.473 [0.396, 0.536]	<.001	a	0.382 [0.311, 0.452]	<.001	a	−0.203 [−0.291,−0.083]	.324
	b	0.381 [0.319, 0.457]	<.001	b	0.294 [0.231, 0.340]	<.001	b	−0.303 [−0.373,−0.193]	.402
HDL‐cholesterol	a	−0.523 [−0.542,−0.422]	<.001	a	−0.321 [−0.442, −0.022]	<.001	a	0.174 [0.152, 0.206]	.051
	b	−0.497 [−0.511, −0.322]	<.001	b	−0.338 [−0.463, −0.029]	<.001	b	0.089 [0.065, 0.118]	<.001
LDL‐cholesterol	a	0.517 [0.406, 0.631]	<.001	a	0.474 [0.386, 0.596]	<.001	a	−0.312 [−0.399,−0.033]	<.001
	b	0.497 [0.364, 0.596]	<.001	b	0.287 [0.239, 0.345]	<.001	b	−0.317 [−0.406, −0.022]	<.001

Abbreviations: a = model non‐adjusted; b = model adjusted for demographic and lifestyle‐related variables; CI = confidence intervals; HDL = high‐density lipoprotein; LDL = low‐density lipoprotein; ORQ = Occupational Role Questionnaire; PRQ = Personal Resources Questionnaire; PSQ = Personal Strain Questionnaire; β = standardized linear regression coefficient.

## 4 DISCUSSION

Previous research in animals and humans has shown that stress is a double‐edged sword, with an “inverted U‐shaped curve” between pressure and performance (McEwen et al., 2015). Stress is initially irritating, but when it exceeds human capacity, there is a turning point (Eysenck, 1985). In our study, we indicated that the OSI‐R scores of medical staff in tertiary public hospitals in Wenzhou were higher than the normative sample because the medical profession is generally considered a very stressful occupation. Although some sources of stress in the healthcare environment are inevitable and constant, some workplace stress factors pose risks to healthcare professionals: such as work organization, financial issues, social life, promotion, relationships with colleagues and patients, and work demands (Tomljenovic et al., 2014). Furthermore, healthcare reform in China, doctors and nurses are under increasing pressure to cure more patients, meet administrative requirements, and keep up with government regulations. In recent years, a few patients who were dissatisfied with treatment have purposely disrupted the normal medical order and posed a threat to the personal safety of medical staff and other patients to seek compensation. Violent incidents have a negative impact on the psychological welfare of healthcare workers (Itzhaki et al., 2015), and their prevalence in China is higher than in other countries (Tiruneh et al., 2016).

Research has documented that emergency service workers show higher levels of stress and health problems than many other occupational groups (Basu et al., 2016). In our study, the emergency department medical staff had high job stress scores and a low ability to response, with high level of serum lipid. Emergency department professionals experience many sources of stress related to both the workload, with the need to perform a large number of tasks and the perception of being under pressure with the requirement to perform tasks very quickly. In addition, doctors and nurses working in the emergency department must deal with the most serious patients in complex situations, such as traffic accidents. Furthermore, unnecessary conflicts may emerge with patients with alcoholism, leading to workplace violence. The current study found that the scores for occupational stresses among male medical staff were higher than those of female medical staff. A possible yet speculative explanation is that men are born with a strong sense of competition, and have relatively large responsibilities in society and family and higher requirements for job title promotion.

In our research, exposure to job stress was found to be significantly associated with dyslipidemia. The blood lipid level of male medical staff was higher than that of female medical staff. Moreover, the level of serum lipids in doctors was higher than that in nurses. Dyslipidemia in the emergency department was more severe than other departments. After controlling demographic and lifestyle‐related variables, occupational stress was positively correlated with dyslipidemia, which supported a past study (Xu et al., 2011). The obtained results indicate the importance of a total OSI and some of its aspects, for the development of dyslipidemia in medical staff through lipid abnormalities. The potential mechanism linking occupational stress and blood lipids is via the hypothalamic–pituitary–adrenal axis (HPA), affecting lipid intake and metabolism (Chida & Steptoe, 2009). Chronic elevations in cortisol levels increase glucocorticoid synthesis and glucose availability and increase visceral fat deposits, lipolysis, and circulating fatty acids (causing associated dyslipidemia). Further study suggests that stress and burnout may influence the incidence of hyperlipidemia by the process of inflammation (Shirom et al., 2013). Moreover, doctors and nurses working in tertiary A public hospitals usually undertake many clinical tasks especially shift work, which is an important part of occupational stress. Shift work was known to be associated with a higher TG/HDL‐C ratio, higher serum TG, and lower HDL‐C levels in a previous study (Alefishat & Abu Farha, 2015). Day and night asymmetry caused by the shift of sleep and food intake time relative to the circadian clock system is thought to contribute to these shift work‐related health issues (Kervezee et al., 2020). One study revealed that the circadian clock orchestrates diverse physiological processes that are critical for health and disease and can affect lipid metabolism (Zheng et al., 2016). The lipid absorption level of intestinal epithelial cells in the clock of mutant mice is significantly higher than that during the day, which leads to higher levels of triglycerides and cholesterol in the body (Gnocchi et al., 2015). Therefore, a reasonable arrangement of shift work may have a positive effect on blood lipid levels in medical staff.

There are a few limitations to this study. First, our study was a cross‐sectional survey and could not provide a causal relationship for the findings of the connection between occupational stress and dyslipidemia. We collected the data through questionnaires, which may lead to potential bias. Second, doctors’ unwillingness to answer questions about personal information such as income made it impossible to explore the effects on occupational stress. Third, this survey was only conducted in tertiary A public hospitals in Wenzhou. There may be important differences in various clinical settings, for instance, work climate, workload, and institutional policy, that limit generalizability. Furthermore, the results were from Wenzhou only and therefore cannot be generalized to all of China. Future studies should include more hospitals and more possible factors.

## 5 CONCLUSIONS

Occupational stress is highly prevalent in the medical profession in Wenzhou. Our study suggests that there is an association between occupational stress and lipid disturbances. Policymakers and hospital administrators should actively intervene in the occupational stress of medical staff, improve the working environment, and help doctors relieve their work stress. Medical staff need to strengthen self‐protection, increase their free time to participate in social activities to reduce stress, and pay attention to health examinations.

## CONFLICT OF INTERESTS

The authors declare that they have no competing interests.

## AUTHOR CONTRIBUTION

Hui‐Zhang collected and analyzed data and wrote the manuscript. Meng‐Meng Shao, Xian‐Da Lin, Li‐Jun Cheng, and Begench Ovlyakulov collected data and conducted the research. Bo‐Bei Chen supervised the design of the experiments and analysis of the data. Ke‐Yang Chen designed conceptualization and critically reviewed the manuscript. All authors read and approved the final manuscript.

### Peer Review

The peer review history for this article is available at https://publons.com/publon/10.1002/brb3.2014.

## Supporting information

Table S1

Table S2

## Data Availability

All the data required for this paper are presented in the manuscript.
