# Supplementary material for: A cross‐sectional survey on occupational stress and associated dyslipidemia among medical staff in tertiary public hospitals in Wenzhou, China
Source: Brain Behav. 2020 Dec 23;11(3):e02014. doi: 10.1002/brb3.2014 (PMC7994692; doi:10.1002/brb3.2014)
Supplement: Supplementary file 1 — Table S1 [file BRB3-11-e02014-s002.docx]

# Post -hoc analysis for Occupational Stress Inventory—revised edition (OSI-R) by different variable

| Variable |  | ORQ | PSQ | PRQ |
| --- | --- | --- | --- | --- |
| Department | Internal | 173.86±19.39 | 100.67±18.87 | 114.57±18.32 |
|  | Surgery | 177.78±17.54 | 102.78±19.52 | 110.49±21.46 |
|  | Pediatrics | 177.63±19.09 | 102.98±16.81 | 109.13±18.76 |
|  | Emergency | 180.03±19.58 | 104.91±17.37 | 108.49±19.11 |
|  | F | 8.29 | 3.85 | 9.18 |
|  | P Value | <0.001 | 0.02 | <0.001 |
|  | Post -hoc analysis |  |  |  |
|  | I VS. S | 0.010 | 0.083 | 0.013 |
|  | I VS. P | 0.018 | 0.048 | 0.000 |
|  | I VS. E | 0.000 | 0.005 | 0.000 |
|  | S VS. P | 0.921 | 0.894 | 0.414 |
|  | S VS. E | 0.143 | 0.053 | 0.133 |
|  | P VS. E | 0.133 | 0.042 | 0.182 |
| Professional title | Junior | 169.53±16.29 | 98.67±17.57 | 114.57±16.34 |
|  | Intermediate | 172.89±17.39 | 100.53±19.12 | 112.96±18.18 |
|  | Associate senior | 173.86±19.53 | 103.67±14.61 | 109.57±20.13 |
|  | Senior | 174.53±15.61 | 103.67±18.87 | 108.38±13.92 |
|  | F | 1.11 | 4.41 | 3.01 |
|  | P Value | 0.428 | 0.010 | 0.063 |
|  | Post -hoc analysis |  |  |  |
|  | J VS. I |  | 0.220 |  |
|  | J VS. AS |  | 0.000 |  |
|  | J VS. S |  | 0.001 |  |
|  | I VS. AS |  | 0.026 |  |
|  | I VS. S |  | 0.046 |  |
|  | AS VS. S |  | 1.000 |  |

I: Internal. P: Pediatrics. E: Emergency. S: Surgery. J: Junior. I: Intermediate.

AS: Associate senior. S: Senior
