# Supplementary material for: A cross‐sectional survey on occupational stress and associated dyslipidemia among medical staff in tertiary public hospitals in Wenzhou, China
Source: Brain Behav. 2020 Dec 23;11(3):e02014. doi: 10.1002/brb3.2014 (PMC7994692; doi:10.1002/brb3.2014)
Supplement: Supplementary file 2 — Table S2 [file BRB3-11-e02014-s001.docx]

Table 3 Comparison of blood lipid levels among multiple variables

| Variables | Department | | | | I.Vs.S | I.Vs.P | I.Vs.E | S.Vs.P | S.Vs.E | P.Vs.E |
| --- | --- | --- | --- | --- | --- | --- | --- | --- | --- | --- |
| blood lipid | Internal | Surgery | Pediatrics | Emergency | P† | P† | P† | P† | P† | P† |
| TG | 1.57±0.95 | 1.60±0.94 | 1.45±0.39 | 1.87±0.47 | 0.700 | 0.046 | 0.000 | 0.012 | 0.000 | 0.000 |
| TC | 4.99±0.85 | 5.10±0.91 | 4.82±0.87 | 5.05±0.97 | 0.130 | 0.017 | 0.425 | 0.000 | 0.519 | 0.003 |
| HDL-C | 1.35±0.33 | 1.32±0.34 | 1.47±0.46 | 1.40±0.35 | 0.278 | 0.000 | 0.075 | 0.000 | 0.005 | 0.038 |
| LDL-C | 2.86±0.71 | 2.89±0.78 | 2.78±0.75 | 2.87±0.82 | 0.626 | 0.185 | 0.874 | 0.082 | 0.762 | 0.165 |
|  | OSI-R group | | | | L.Vs.M | L.Vs.H | M.Vs.H |  |  |  |
|  | Low OSI-R | Moderate OSI-R | High OSI-R |  | P† | P† | P† |  |  |  |
| TG | 1.33±0.53 | 1.72±0.49 | 1.74±0.39 |  | 0.000 | 0.000 | 0.527 |  |  |  |
| TC | 4.61±0.85 | 5.18±1.54 | 5.89±1.13 |  | 0.000 | 0.000 | 0.000 |  |  |  |
| HDL-C | 1.43±0.42 | 1.32±0.21 | 1.29±0.28 |  | 0.000 | 0.000 | 0.090 |  |  |  |
| LDL-C | 2.73±0.68 | 2.56±0.64 | 2.35±0.59 |  | 0.000 | 0.000 | 0.000 |  |  |  |

I: Internal. P: Pediatrics. E: Emergency. S: Surgery. †Tukey’s Honest Significant Difference post hoc analysis.
